# Supplementary material for: Leveraging mHealth usage logs to inform health worker performance in a Resource-Limited setting: Case example of mUzima use for a chronic disease program in Western Kenya
Source: PLOS Digit Health. 2022 Sep 1;1(9):e0000096. doi: 10.1371/journal.pdig.0000096 (PMC9931325; doi:10.1371/journal.pdig.0000096)
Supplement: S1 Appendix — (DOCX) [file pdig.0000096.s001.docx]

| EVENT LOG FIELD | DESCRIPTION | AVAILABILITY |
| --- | --- | --- |
| User ID | Machine generated unique identifier for the health worker interacting with the mobile application. | Mandatory for all types of usage logs |
| Patient ID | Machine generated unique identifier for the patient being seen by the health worker | Mandatory for all types of usage logs related to this study |
| Device ID | Machine generated unique identifier for the mobile device being used by the health worker | Mandatory for all types of usage logs |
| Tag | A unique identifier for types of usage logs generated by the mobile application. The various tags for all events logged by the app are provided in Appendix 8. | Mandatory for all types of usage logs |
| Event timestamp | The timestamp of when the event happened, determined by the time settings of the mobile device | Mandatory for all types of usage logs |
| Transmission timestamp | The timestamp of when the usage log was received at the log server, as determined by the time settings of the log server | Available only when GPS location information is available, otherwise it will have the value *NA* |
| GPS timestamp | The timestamp of when the most recent GPS location of the mobile device was recorded relative to when the event happened, as determined by the mobile device time settings. | Available only when GPS location information is available, otherwise it will have the value *NA* |
| GPS longitude | The longitude of the most recent GPS location of the mobile device relative to when the event happened | Available only when GPS location information is available, otherwise it will have the value *NA* |
| GPS latitude | The longitude of the most recent GPS location of the mobile device relative to when the event happened | Available only when GPS location information is available, otherwise it will have the value *NA* |
| GPS accuracy | The accuracy of the most recent GPS location of the mobile device relative to when the event happened | Available only when GPS location information is available, otherwise it will have the value *NA* |
| GPS provider | The source of the GPS location information. Values for this field will be either of the following:   - *GPS* : The GPS location sensor on the mobile device that uses location satellites to provide more accurate GPS information, but is slower and consumes more power - *Network*: Determines GPS location based on cell tower and WiFi access points. Results are retrieved by means of a network lookup. This is mostly accurate but is faster and consumes lee power | Available only when GPS location information is available |
| GPS location status | The status of GPS location settings on the mobile device. Values can be interpreted as follows:   - *Available*: This is when location settings are enabled and the location information was availed - *Location unavailable*: This is when the application was granted permissions to use GPS location feature, but the user did not turn on location settings on the device, and therefore GPS location information was not availed - *Permission not granted by User*: This is when the user did not grant permissions for the mobile application to access GPS location feature. | This information is available for all logs |
| Recorded data ID | A machine generated unique identifier for identifying/tracing patient data recorded by the health worker for events related to opening and saving data collection forms. | This is available only for events related to opening and filling of forms. |
