# Supplementary material for: Leveraging mHealth usage logs to inform health worker performance in a Resource-Limited setting: Case example of mUzima use for a chronic disease program in Western Kenya
Source: PLOS Digit Health. 2022 Sep 1;1(9):e0000096. doi: 10.1371/journal.pdig.0000096 (PMC9931325; doi:10.1371/journal.pdig.0000096)
Supplement: S2 Appendix — (DOCX) [file pdig.0000096.s002.docx]

| LOG TYPE IDENTIFIER (TAG) | DESCRIPTION |
| --- | --- |
| VIEW_CLIENT_LIST | This is generated when the user navigates to the page showing the list of client records available on the mobile device. |
| VIEW_PATIENT_REMOTE_SEARCH | This is generated when the user hits the button to search for a client record against the server, so that if found, the record may be downloaded to the mobile device. |
| VIEW_CLIENT_SUMMARY | This is generated when the user navigates to the client summary page of the mobile application by selecting the client record from the list available on the page tagged VIEW_CLIENT_LIST.  From this page, the user can navigate further to access observations, encounters, forms, notifications and clinical summary. |
| VIEW_CLIENT_OBS_BY_CONCEPT | This is generated when the user navigates to the page for viewing list of observations recorded for the patient historically, grouped by type of observation (concept/terminology) |
| VIEW_CLIENT_OBS_BY_DATE | This is generated when the user navigates to the page for viewing the list of observations recorded for the patient historically, grouped by date of when the observation happened. |
| VIEW_PATIENT_NOTIFICATIONS | This is generated when the user navigates to the page for viewing the list of notifications/messages related to a patient |
| VIEW_CLIENT_ENCOUNTERS | This is generated when the user navigates to the page for viewing list of historical encounters recorded for the patient |
| VIEW_ENCOUNTER_SUMMARY | This is generated when the user navigates to the page for viewing list of observations belonging to a selected encounter recorded for the patient historically |
| VIEW_CLIENT_DOWNLOADED_REPORTS | This is generated when the user navigates to the page for viewing list of a patient’s clinical summary reports that have been downloaded to the mobile device |
| VIEW_CLIENT_ALL_REPORTS | This is generated when the user navigates to the page for viewing the list of a patient’s available clinical summary reports inclusive of those that have been downloaded as well as those not downloaded to the mobile device |
| VIEW_RECOMMENDED_FORMS | This is generated when the user navigates to the page for viewing the list of encounter forms available for recording patient data.  The user reaches this page by selecting the tab named forms available on the client summary page (tag VIEW_CLIENT_SUMMARY). |
| VIEW_INCOMPLETE_PATIENT_FORMS | This is generated when the user navigates to the page for viewing the list of forms that were saved as incomplete.  The page is available as a second section to the page tagged VIEW_RECOMMENDED_FORMS. |
| VIEW_COMPLETED_PATIENT_FORMS | This is generated when the user navigates to the page for viewing the list of forms that were saved as complete.  The page is available as a third section to the page tagged VIEW_RECOMMENDED_FORMS. |
| OPEN_ENCOUNTER_FORM | This is generated when the health worker opens an encounter form to start recording data for a patient.  The form is opened by selecting one of the items listed under the page tagged VIEW_RECOMMENDED_FORMS. |
| OPEN_REGISTRATION_FORM | This is generated when the health worker opens a registration form for a patient.  The user navigates to the client/patient list page to reach the page to initiate new patient registration |
| RESUME_ENCOUNTER_FORM | This is generated when the health worker reopens an encounter form that had previously been saved.  The user navigates through any of the pages tagged VIEW_INCOMPLETE_PATIENT_FORMS, VIEW_COMPLETE_PATIENT_FORMS, VIEW_INCOMPLETE_FORMS or VIEW_COMPLETE_FORMS |
| RESUME_REGISTRATION_FORM | This is generated when the health worker reopens a registration form that had previously been saved.  The user navigates through any of the pages tagged VIEW_COMPLETE_PATIENT_FORMS, VIEW_INCOMPLETE_FORMS or VIEW_COMPLETE_FORMS |
| SAVE_DRAFT_ENCOUNTER_FORM | This is generated when the user saves an encounter form as draft with intention to reopen and complete filling the form later. |
| SAVE_DRAFT_REGISTRATION_FORM | This is generated when the user saves an encounter form as draft with intention to reopen and complete filling the form later. |
| SAVE_COMPLETE_ENCOUNTER_FORM | This is generated when the health worker saves an encounter form as complete. At this point, the saved form data can be transmitted to the EMR. |
| SAVE_COMPLETE_REGISTRATION_FORM | This is generated when the health worker saves a registration form as complete. At this point, the saved form data can be transmitted to the EMR. |
| FORM_CLOSED | This is generated when the user exits a form that had been opened, by hitting the back button that triggers navigation to the previous screen, or after hitting the save button that triggers saving of the form data and subsequently closing of the form view. |
| OPEN_FORM | For a new version of the mobile application, the OPEN_ENCOUNTER_FORM and the OPEN_REGISTRATION_FORM tags have been combined into the OPEN_FORM tag, with an additional parameter (form discriminator) used to describe the type of form |
| SAVE_FORM | For a new version of the mobile application, the SAVE_ENCOUNTER_FORM and the SAVE_REGISTRATION_FORM tags have been combined into the SAVE_FORM tag, with an additional parameter (form discriminator) used to describe the type of form |
| SAVE_DRAFT_FORM | For a new version of the mobile application, the SAVE_DRAFT_ENCOUNTER_FORM and the SAVE_DRAFT_REGISTRATION_FORM tags have been combined into the SAVE_DRAFT_FORM tag, with an additional parameter (form discriminator) used to describe the type of form |
| RESUME_FORM | For a new version of the mobile application, the RESUME_ENCOUNTER_FORM and the RESUME_REGISTRATION_FORM tags have been combined into the RESUME_FORM tag, with an additional parameter (form discriminator) used to describe the type of form |
| CDM_REMINDERS | This is generated based on logic programmed in a data collection form used via the mobile app. |
| CDM_REMINDER_RESPONSE | This is generated based on logic programmed in a data collection form used via the mobile app. |
| VIEW_DASHBOARD | This is generated when the user visits the app *dashboard* page, in order to access tabs representing key app features. |
| VIEW_ALL_FORMS | This is generated when the user navigates to the page showing the list of names for data collection forms available in the EMR, including those that are already downloaded to the mobile device. The user hits the *dashboard* tab labeled *Forms* in order get to this page. |
| VIEW_DOWNLOADED_FORMS | This is generated when the user navigates to the page showing the list of names for data collection forms available in the EMR and already downloaded to the mobile device. This is available as a second section to the page tagged *VIEW_ALL_FORMS*. |
| VIEW_COMPLETED_FORMS | This is generated when the user navigates to the page showing the list of names for data items collected via the app and saved as *draft*. This is available as a third section to the page tagged VIEW_ALL_FORMS. |
| VIEW_INCOMPLETE_FORMS | This is generated when the user navigates to the page showing the list of names for data items collected via the app and saved as *completed*. This is available as a third section to the page tagged VIEW_ALL_FORMS. |
| VIEW_ALL_COHORTS | This is generated when the user navigates to the page showing the list of names for patient groups available in the EMR, including those that are already downloaded to the mobile device. The user hits the dashboard tab labeled *Cohorts* in order get to this page. |
| VIEW_SYNCED_COHORTS | This is generated when the user navigates to the page showing the list of names for patient cohorts available in the EMR and already downloaded to the mobile device. This is available as a second section to the page tagged *VIEW_ALL_COHORTS*. |
| VIEW_FORM_DOWNLOAD_WIZARD | This is generated when the user navigates to the page for downloading data collection forms. |
| VIEW_LOCATION_DOWNLOAD_WIZARD | This is generated when the user navigates to the page for downloading names and other details of facilities when data collection is to happen. |
| VIEW_PROVIDER_DOWNLOAD_WIZARD | This is generated when the user navigates to the page for downloading names and other details of health workers who use the device for data collection via the app. |
| VIEW_COHORT_DOWNLOAD_WIZARD | This is generated when the user navigates to the page for downloading names and other details of patient cohorts available on the EMR. |
| VIEW_CONCEPT_DOWNLOAD_WIZARD | This is generated when the user navigates to the page for downloading names and other details of medical terminologies whose related patient data is downloaded from the EMR or retained on the device when recorded via the app. |
| VIEW_LOCATION_PREFERENCE | This is generated when the user navigates to the page for downloading names and other details of facilities when data collection is to happen. |
| VIEW_PROVIDER_PREFERENCE | This is generated when the user navigates to the page for downloading additional names and other details of health workers who use the device for data collection via the app. |
| VIEW_CONCEPT_PREFERENCE | This is generated when the user navigates to the page for downloading additional names and other details of medical terminologies whose related patient data is downloaded from the EMR or retained on the device when recorded via the app. |
| VIEW_SETTINGS | This is generated when the when the user navigates to the page showing available settings for customizing the application based on user or implementation preferences. |
| VIEW_LOCATIONS | This is generated when the user navigates to the page for downloading additional names and other details of facilities when data collection is to happen. |
| VIEW_SETUP_METHODS | This is generated when the user navigates to the page showing available options for setting up the app for use, which happens when the app is being used for the first time after installation or after resetting it via *Android* mobile application settings. |
| VIEW_GUIDED_SETUP_METHOD | This is generated when the user navigates to the page where the app automatically performs the initial setup as per resource settings defined in the selected configuration, when the guided setup method is selected. |
| VIEW_PROVIDER_LIST | This is generated when the user navigates to the page for downloading additional names and other details of health workers who use the device for data collection via the app. |
| VIEW_CONCEPT_LIST | This is generated when the user navigates to the page for downloading names and other details of medical terminologies whose related patient data is downloaded from the EMR or retained on the device when recorded via the app. |
| VIEW_COHORT_PREFERENCE | This is generated when the user navigates to the page for specifying preferences on the kind of cohort. |
| VIEW_GENERAL_NOTIFICATIONS | This is generated when the user navigates to the page showing the list of messages sent to the current user by other users via the EMR. The user hits the dashboard tab labeled *Notifications* in order get to this page. |
| VIEW_HELP | This is generated when the user navigates to the page showing user guides. |
| VIEW_PROVIDER_REPORTS | This is generated when the user navigates to the page showing reports. |
| VIEW_FEEDBACK_PAGE | This is generated when the user navigates to the page used to submit communication to developers of the application such as comments. |
| UPLOAD_FORM_DATA | This is generated when the user hits the button to submit form data collected on the device to the EMR. |
| SYNCED_FORM_DATA | This is generated when form data collected on the device has been successfully submitted to the EMR. This applies to both situations when the user triggers the data upload or when the app automatically submits the data. |
| FORM_DATA_PROCESSED_SUCCESSFULLY | This is generated when the app polls the EMR to check whether submitted data was successfully processed and receives a positive confirmation. |
| LOGIN_FAILURE | This is generated when authentication of user credentials supplied at the login page fails. |
| LOGIN_SUCCESS | This is generated when authentication of user credentials supplied at the login page is successful. |
| SESSION_TIMEOUT | This is generated when the user does not use the app for some time and the app automatically terminates the authenticated session. By default, the session is timed out after five minutes. |
| USER_LOGOUT | This is generated after the user is logged out from the app by selecting the option to logout from the app menu or by hitting the back button while on the page tagged VIEW_DASHBOARD. |
