# Supplementary figures and images for: Leveraging mHealth usage logs to inform health worker performance in a Resource-Limited setting: Case example of mUzima use for a chronic disease program in Western Kenya
Source: PLOS Digit Health. 2022 Sep 1;1(9):e0000096. doi: 10.1371/journal.pdig.0000096 (PMC9931325; doi:10.1371/journal.pdig.0000096)

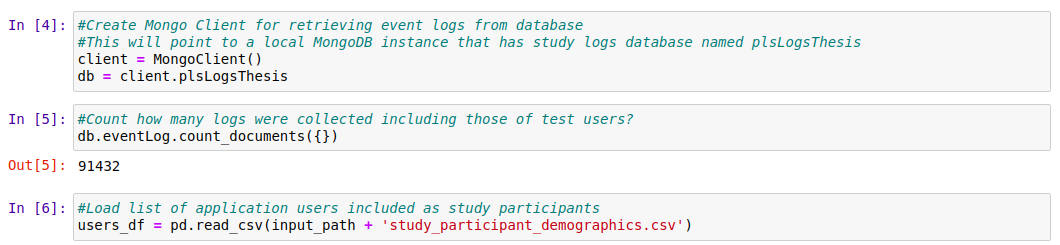


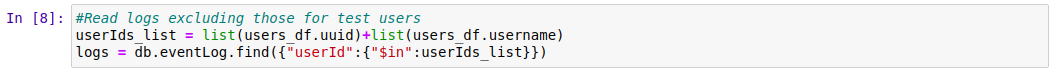


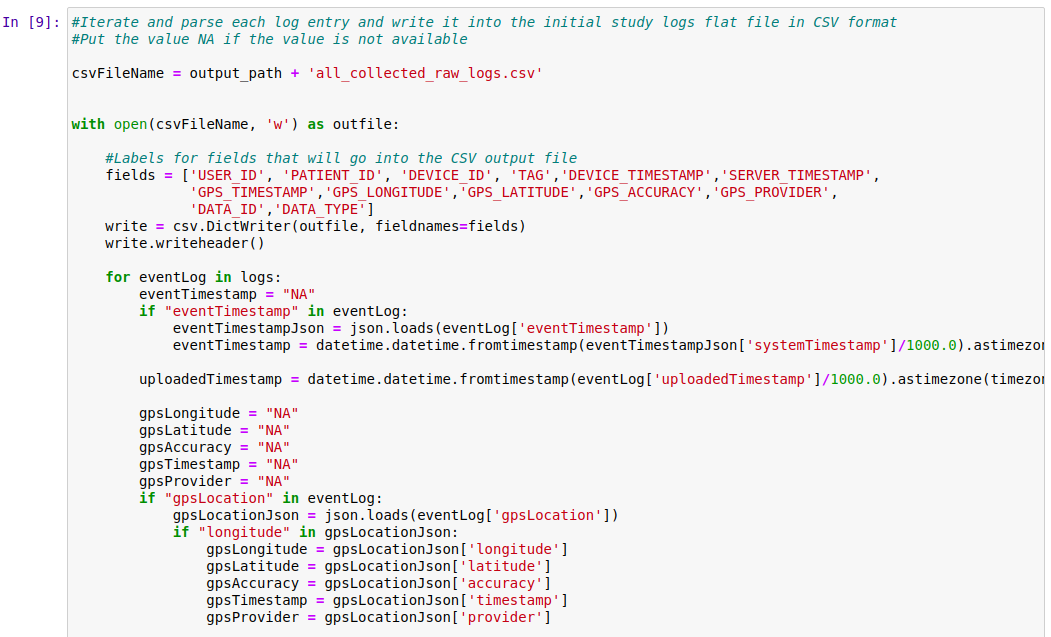


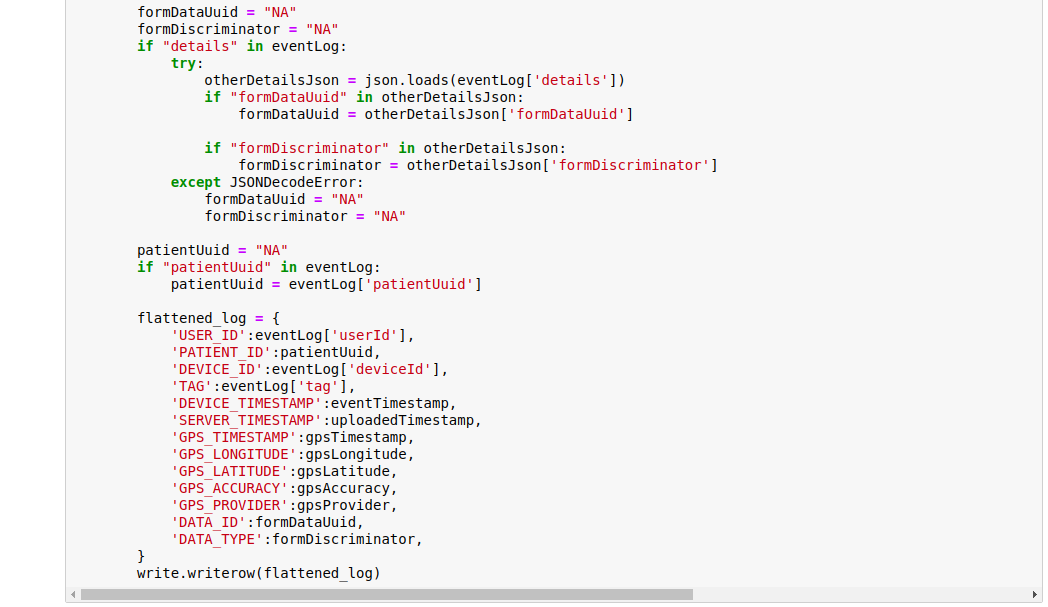


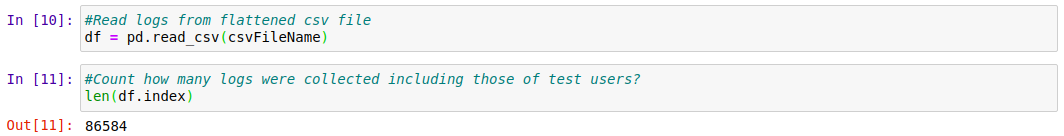


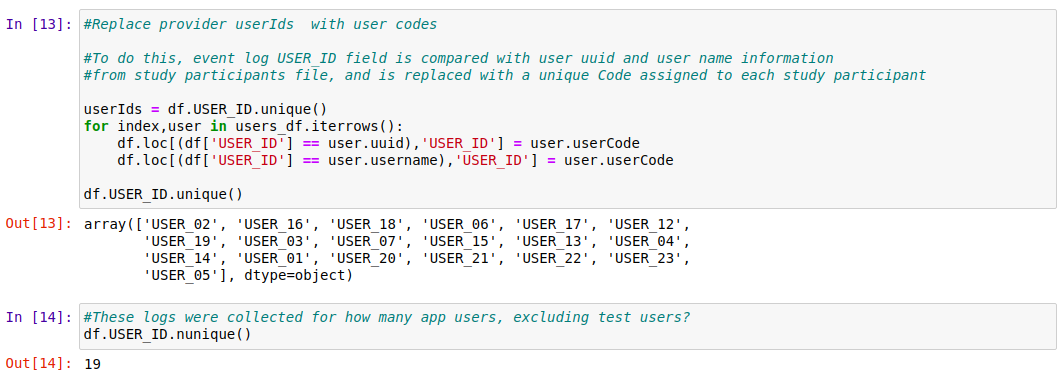


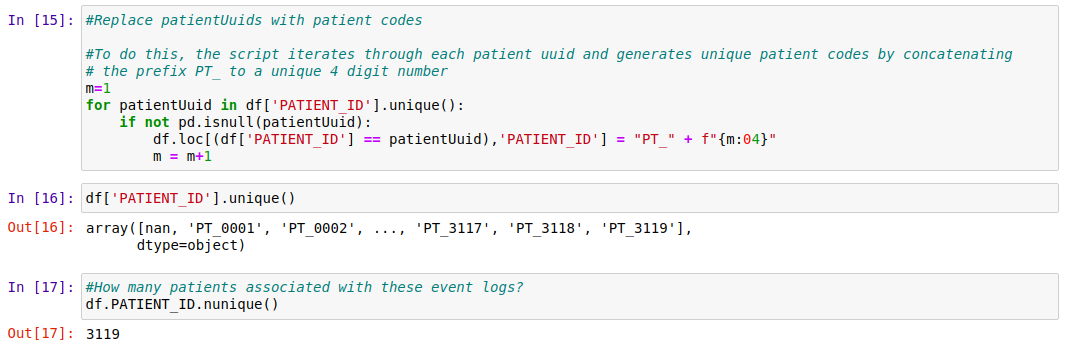


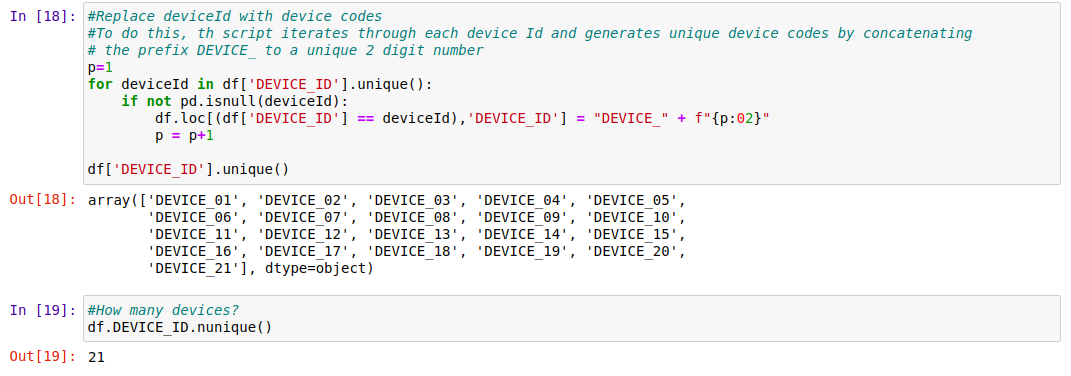


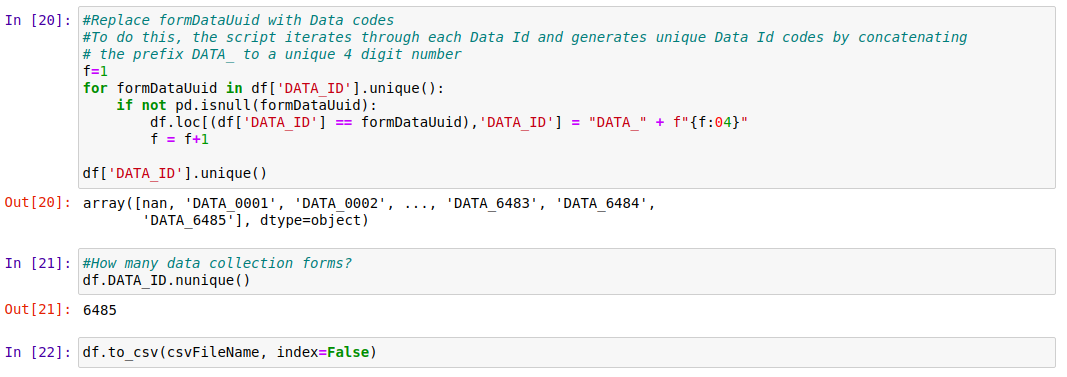


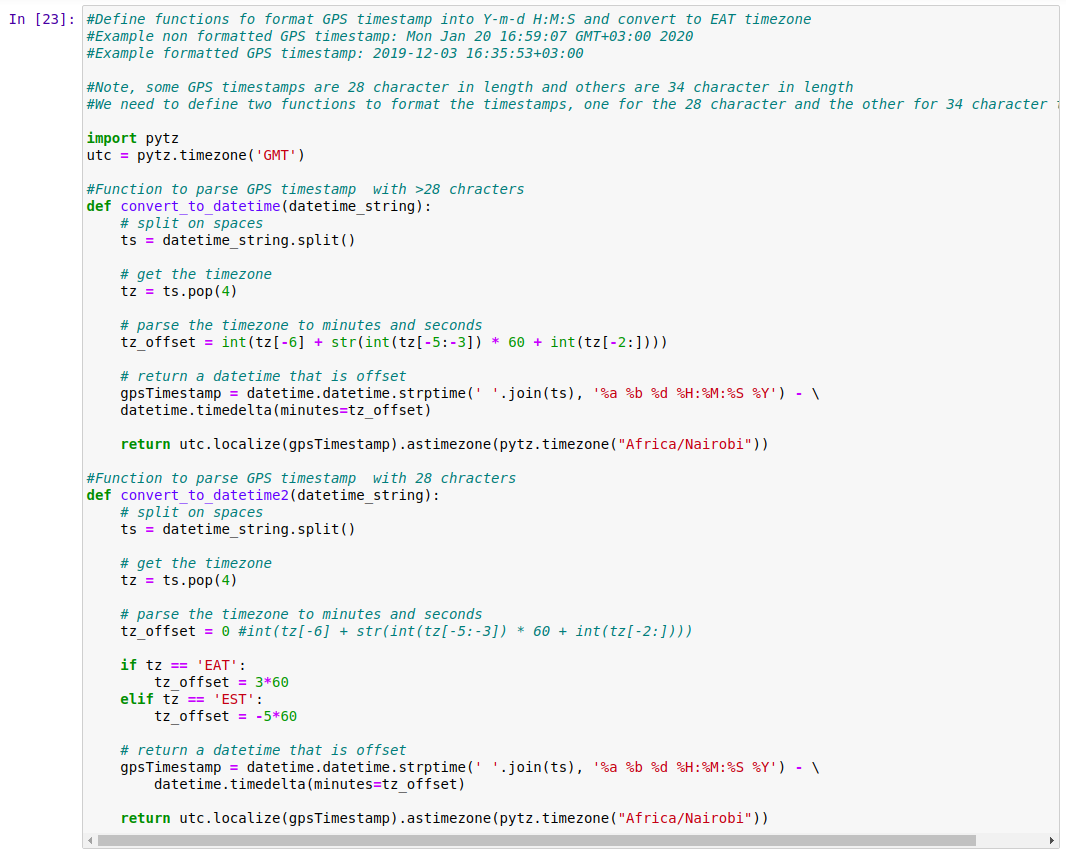


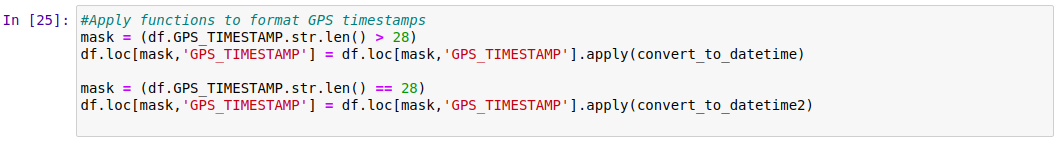

Supplement: S3 Appendix — (DOCX) [file pdig.0000096.s003.docx]

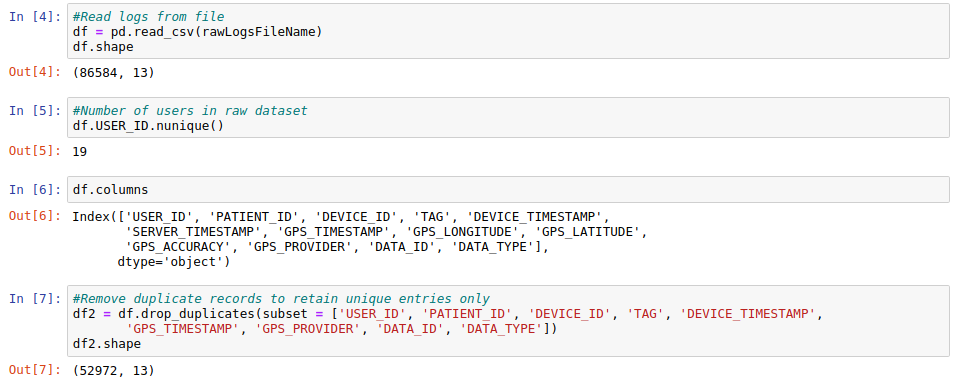


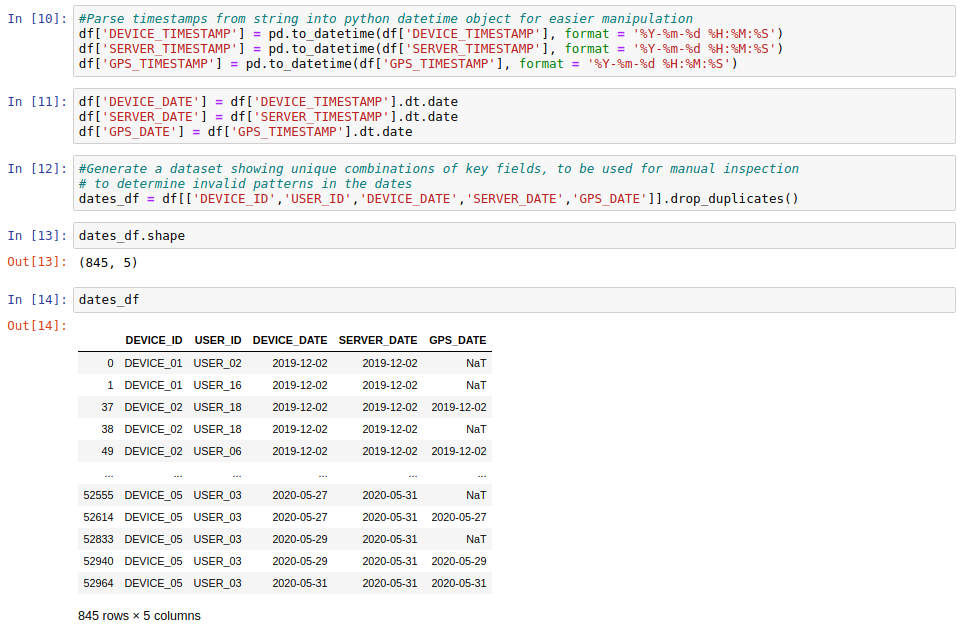


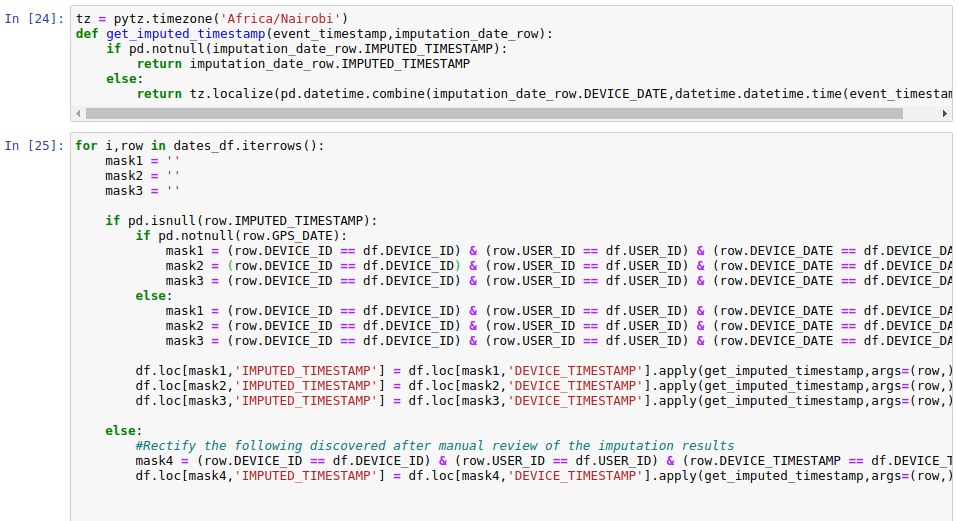

Supplement: S4 Appendix — (DOCX) [file pdig.0000096.s004.docx]

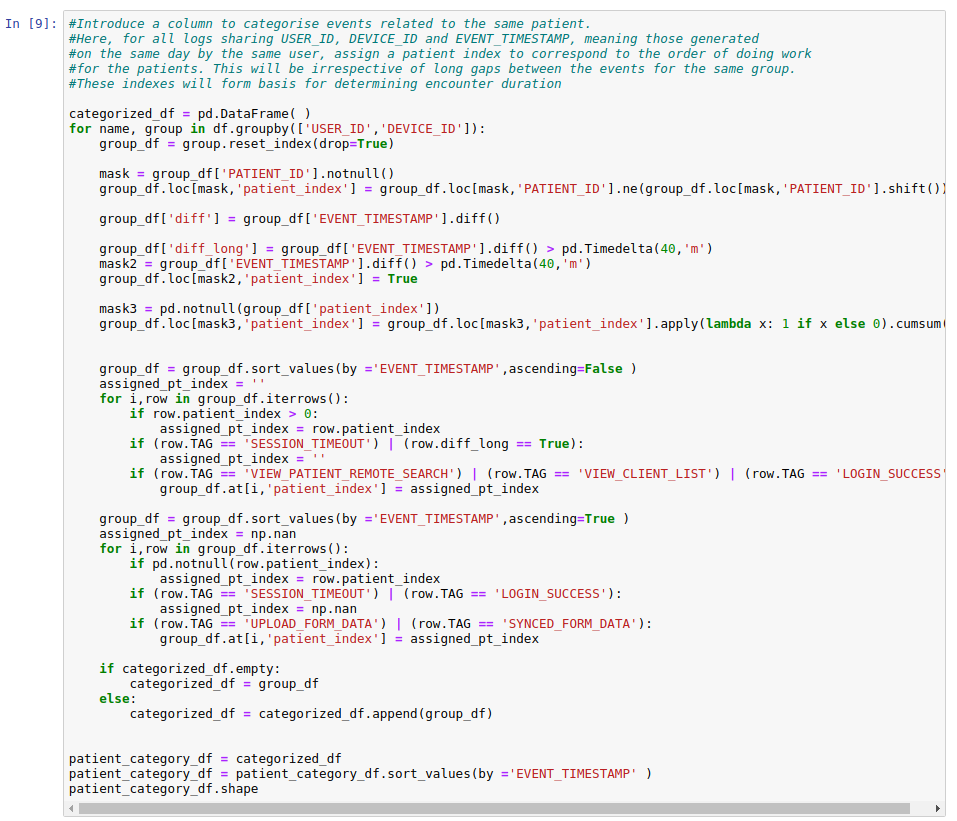

Supplement: S5 Appendix — (DOCX) [file pdig.0000096.s005.docx]
