# Supplementary material for: Leveraging mHealth usage logs to inform health worker performance in a Resource-Limited setting: Case example of mUzima use for a chronic disease program in Western Kenya
Source: PLOS Digit Health. 2022 Sep 1;1(9):e0000096. doi: 10.1371/journal.pdig.0000096 (PMC9931325; doi:10.1371/journal.pdig.0000096)
Supplement: Attachment — Submitted filename: 20220627 PLoS Digital Response Cover Letter.docx [file pdig.0000096.s006.docx]

**Re: Manuscript ID #PDIG-D-22-00108 - *Leveraging mHealth Usage Logs to Inform Health Worker Performance in a Resource-Limited Setting: Case Example of mUzima use for a Chronic Disease Program in Western Kenya*.**

We appreciate the review by PLOS Digital Health of our manuscript entitled "*Leveraging mHealth Usage Logs to Inform Health Worker Performance in a Resource-Limited Setting: Case Example of mUzima use for a Chronic Disease Program in Western Kenya*.” We are grateful for the reviewers’ inputs and appreciate the opportunity to respond comprehensively to the comments.

Please find our responses to all the comments by the reviewers below.

**1.** Please amend your detailed Financial Disclosure statement. This is published with the article. It must therefore be completed in full sentences and contain the exact wording you wish to be published.

Please state what role the funders took in the study. If the funders had no role in your study, please state: “The funders had no role in study design, data collection and analysis, decision to publish, or preparation of the manuscript.”

**Response:** We have made this amendment in the Financial Disclosure statement which now states: *“The funders had no role in study design, data collection and analysis, decision to publish, or preparation of the manuscript.*”

**2.** Please send a completed 'Competing Interests' statement, including any COIs declared by your co-authors. If you have no competing interests to declare, please state "The authors have declared that no competing interests exist". Otherwise please declare all competing interests beginning with the statement "I have read the journal's policy and the authors of this manuscript have the following competing interests:"

**Response:** We have made this amendment in the 'Competing Interests' statement. It now states: *"The authors have declared that no competing interests exist".*

3. In the online submission form, you indicated that "Data will be availed with appropriate approvals.". All PLOS journals now require all data underlying the findings described in their manuscript to be freely available to other researchers, either 1. In a public repository, 2. Within the manuscript itself, or 3. Uploaded as supplementary information.

This policy applies to all data except where public deposition would breach compliance with the protocol approved by your research ethics board. If your data cannot be made publicly available for ethical or legal reasons (e.g., public availability would compromise patient privacy), please explain your reasons by return email and your exemption request will be escalated to the editor for approval. Your exemption request will be handled independently and will not hold up the peer review process, but will need to be resolved should your manuscript be accepted for publication. One of the Editorial team will then be in touch if there are any issues. I think we can avail de-identified data. Needs discussion on the best channels to use.

**Response**: Data will be uploaded as supplementary information.

4. Please provide separate figure files in .tif or .eps format and remove any figures embedded in your manuscript file. Please also ensure that all files are under our size limit of 10MB.

For more information about how to convert your figure files please see our guidelines: [https://nam12.safelinks.protection.outlook.com/?url=https%3A%2F%2Fjournals.plos.org%2Fdigitalhealth%2Fs%2Ffigures&amp;data=05%7C01%7Cmartin.c.were%40vumc.org%7C17f2c03c66794a89e36408da54bed8a0%7Cef57503014244ed8b83c12c533d879ab%7C0%7C0%7C637915475899617807%7CUnknown%7CTWFpbGZsb3d8eyJWIjoiMC4wLjAwMDAiLCJQIjoiV2luMzIiLCJBTiI6Ik1haWwiLCJXVCI6Mn0%3D%7C3000%7C%7C%7C&amp;sdata=49OtgSu5XacLXTOrago%2FzN6SIfNsLzOxQYZLpqsTmAQ%3D&amp;reserved=0](https://nam12.safelinks.protection.outlook.com/?url=https%3A%2F%2Fjournals.plos.org%2Fdigitalhealth%2Fs%2Ffigures&data=05%7C01%7Cmartin.c.were%40vumc.org%7C7c0aea64b4d54a96666208da64b82d93%7Cef57503014244ed8b83c12c533d879ab%7C0%7C0%7C637933039454368862%7CUnknown%7CTWFpbGZsb3d8eyJWIjoiMC4wLjAwMDAiLCJQIjoiV2luMzIiLCJBTiI6Ik1haWwiLCJXVCI6Mn0%3D%7C3000%7C%7C%7C&sdata=4iovZx3OfKlzMR1B4HZOokvpOhyJhDhyyHeuqmR9nfg%3D&reserved=0)

**Response**: We have provided separate figure files in .tif format, and removed any figures embedded in the manuscript. All files are under 10 MB in size.

5. We do not publish any copyright or trademark symbols that usually accompany proprietary names, eg (R), (C), or TM (e.g. next to drug or reagent names). Please remove all instances of trademark/copyright symbols throughout the text, including © World Bank on page 25.

**Response**: We have removed all copyright and trademark symbols.

6. Please review your reference list to ensure that it is complete and correct. If you have cited papers that have been retracted, please include the rationale for doing so in the manuscript text, or remove these references and replace them with relevant current references. Any changes to the reference list should be mentioned in the rebuttal letter that accompanies your revised manuscript. If you need to cite a retracted article, indicate the article's retracted status in the References list and also include a citation and full reference for the retraction notice.

**Response:** All references have been reviewed and updated. There are no retracted papers among the references.

Additional Editor Comments (if provided):

REVIEWER’S COMMENTS:

**Reviewer #1:**

Comment 1: The authors report the results of a study conducted at a chronic disease program in Kenya. The study participants used an mHealth application (mUzima) during clinical care. In addition to having a decision support system, the app also captured usage logs. The objective of this study was to valuate usefulness of mHealth usage logs to inform health worker performance.

I find the paper interesting and useful. However, I believe the paper is incomplete. Although the authors’ stated objective is to valuate usefulness of mHealth usage logs to inform health worker performance, they did not achieve that.

After presenting the data analysis, the authors leave it to future work to reasons of the findings and how the findings could be used to improve the health delivery process. (they state in lines 345-351 that “As the next steps, we will employ qualitative approaches to better understand reasons for the observed work patterns and for the variations in performance between providers. We will also leverage the paradata-derived metrics to provide real-time visualization to providers for insights on their own individual performance, and their performance.”) Please note that the study was completed more than two years ago. Although the results are generalizable, results would be valuable as a case study if they could explain how and why they did or did not use the findings to improve the care delivery process.

**Response:** We appreciate the reviewer’s comment and agree that it is useful to conduct qualitative analyses to better understand reasons for the observed work patterns as an extension to this study. While qualitative assessment is planned, events related to personnel changes, the COVID-19 pandemic and funding constraints have delayed this qualitative component. The team is working to address these challenges for this separate qualitative study. Of note, findings from the study have been shared with the clinical team specifically to guide work attendance and work hours. We have now included this in the manuscript: “*Results of this study have been shared with the clinical team to help inform work patterns and approaches for improving them*.” (*Discussion: Last paragraph*)

The question this study was primarily asking was whether mHealth usage logs can be used to inform health worker performance, and these are the results we have reported in the current paper. We agree that explanations for the observed worker patterns should be evaluated as suggested by the reviewer.

Comment 2: In addition to this, it would be useful if they provide the following information:

What are the work requirements for each worker? Do the workers use the app only outside of their facilities? It is mentioned (line 229) that in total, 39,229 log records were collected for the 13 study participants, and these included 2,497 clinical encounters. It does not mention how many workdays are included in this three-months study period. My rough calculation leads to 115 workdays per worker giving a total of about 1485 workdays. However, from figure 5, total number of resulting workdays is only about 160 workdays, which is only 10% of the workdays. It is important that the authors justify this.

**Response:** We appreciate the reviewer’s comment. Official work hours are usually from 8 am to 5 pm from Monday to Friday. Part of the reason for this study was to highlight compliance with these outlined hours and days. It should be noted that there were also no clear workday schedules for the individuals involved in the study, and individuals took two weeks off around the Christmas and New Years holidays. Even with these, the total number of days worked with use of the mobile application is still low, pointing towards potential absenteeism from work. We have now included the following in the manuscript:

*“Within the study setting official clinician work hours were between 8 am to 5 pm from Monday to Friday.*” (Methods: Study Setting) and "*At the time of the study, no clear workday schedules were available for the clinicians, and individuals took two weeks off around the Christmas and New Years holidays*.” (Methods: Study Procedures)

Comment 3: Based on figure 3, newly collected patient data from the app is automatically uploaded to EMR. If so, the correlation coefficient should be 1, not 0.92.

**Response:** Thanks for this comment and sorry for not being clear. Within the mobile device, providers could occasionally start an encounter / visit, but not complete it. These would not be uploaded to the EMRs until the incomplete forms were completed - which sometimes was on a different day. We counted days worked to include both days where the providers used the mobile form to collect patient information, even when they were incomplete. As such, days worked, based on log data was a bit higher, hence the correlation coefficient of 0.92. However, if we only compare days from the log data with complete encounters, it matched that in the EMRs for a correlation coefficient of 1. We have updated the relevant paragraph as follows:

*“Comparison of the total numbers of the work days for each participant as derived from the logs as well as from records in the EMRs showed a strong positive correlation between the two data sources (r(11)=.92, p<.0005). Workdays using mobile logs also included days where clinicians used mUzima to fill an encounter form but did not complete the form – these incomplete forms were not transmitted to the EMRs until they were completed, hence work days were higher from log-based data when compared to EMRs data. A workday was defined as a unique date with evidence of logs related to a patient clinical encounter.”* (Results: Distribution of Work Activity by Number of Days Worked)

Comment 4: Figure 6 supersedes figure 4 and hence delete Figures 4. Also, it is not necessary to present user numbers sequentially. A Pareto chart is much more meaningful here.

**Response**: We agree with the reviewer and have deleted Figure 4 and provided a Pareto chart, arranged by number of days worked.

Comment 5: Figures 8 and 9 are not informative as presented. If the objective is to visualize the variation in length of workdays, a simple relative frequency distribution of length of workdays would be useful. Note that the actual dates are unimportant. You can also have two relative frequency distributions in figures 8 and 9 in one figure.

**Response**: We appreciate the reviewer’s comment. The objective of these figures was to show the variation in start and end time for a work day for the clinicians, especially when compared with the government-mandated work hours of 8 am to 5 pm. Figure 9 simply provides a visualization for one of the study participants to easily highlight and demonstrate how patterns of work can be derived from logs for each clinician.

**Reviewer #2**:

Comment 1: The authors present a novel use of usage log data to track health provider activity (a surrogate for performance) while using a mobile application (mUzima) that is linked to a larger electronic medical record system within the primary health care system in several counties in Western Kenya. The manuscript is well written, and the authors have provided adequate information regarding their methods and analyses. The additional appendices were also helpful to understand the scope of the data they gathered from the system for their research.

The authors conclude that they were able to reliably demonstrate that the log data can provide detailed information of the days and hours worked, number of patients seen per day, and how the application was being used to inform many areas of improvement related to health worker performance. They also observed large differences in work performance between health providers, and work performed outside of official work hours and on weekends and the authors also determined that many (if not all) of the health providers also used the application sub-optimally for entering patient data after the patient visit, as opposed to using the application during the patient encounter to best leverage built-in clinical decision support functionality.

**Response**: The comments by the reviewer are appreciated.

Comment 2: The research would have been strengthened by using a mixed-methods approach to also interview the providers who participated in the study to better understand what factors impacted their work performance (barriers such as workload, lack of access to the mobile network/mobile data credit to utilize the application in real time, and/or functionality of the mUzima application itself etc), however, the COVID-19 pandemic may have made this complementary data collection possible (physical distancing etc) as the study data was collected in the 3 months leading up to the declaration of the global pandemic (March 2020). The authors state in their discussion that they recognize that the lack of qualitative data is a weakness/limitation and that they plan to explore qualitative approaches to better understand their research findings.

**Response**: We agree with the reviewer that a qualitative component is needed, and this is planned. A response to a similar component from Reviewer #1 is available under Comment #1.

Comment 3: The authors used the usage log data as a surrogate for ‘work performance’. While this is a reasonable surrogate to track clinical activities (as mUzima links to the EMR) and using the usage log data can serve as a surrogate for work performance, they are not entirely equal. The authors have addressed this adequately within their discussion and they have also highlighted the ethical implications of using these types of approaches strictly to inform, support, and incentivize care providers and not use the data for punitive purposes.

**Response**: Thank you for this comment.

Comment 4: Overall, the research is novel and serves as an excellent jumping off point for further research in this area. It has significant potential to be utilized and leveraged further for future efforts and strategies to improve health worker performance and strengthen the use of mHealth approaches for health system strengthening, especially within chronic disease management in primary care.

**Response**: We appreciate the reviewer’s comments and encouragement on this work.

Comment 5: I did note one error/oversight in the reference list. Reference #13 and #35 are the same reference. This needs to be corrected.

**Response**: We apologize for this oversight. The duplicate reference has been removed and all references updated.

**General Comment:** While revising your submission, please upload your figure files to the Preflight Analysis and Conversion Engine (PACE) digital diagnostic tool.

**Response**: We have ensured all figures comply with the PLOS requirements by uploading them in the Preflight Analysis and Conversion Engine (PACE) digital diagnostic tool.

We truly appreciate the feedback from the reviewers which have strengthened our manuscript. We hope that you find our responses satisfactory, and thank you again for considering our manuscript for PLOS Digital Health.

Sincerely,

Martin C. Were, MD MS

martin.c.were@vumc.org
